# Controlling a Hypersensitive Palate When Taking Impressions

**Published:** 1907-04

**Authors:** A. E. Franklin


### CONTROLLING A HYPERSENSITIVE PALATE WHEN TAKING IMPRESSIONS.

A gentleman, about sixty years of age, called at my office, claiming that he had been unable to get a set of teeth because no dentist had been able to get the impressions, his throat and palate being so sensitive. The last dentist he had visited, after trying cocaine as a spray, and various other methods, told him to go home and tickle his throat with a long feather. This he did, with the result that his stomach and nervous system were in a very bad condition when he applied to me. I was once advised by a physician to use chloretone in such cases. After giving the man the following doses of chloretone, I was enabled to take my impressions with no unpleasant symptoms whatever. I gave him three powders of chloretone, each containing five grains, and directed him to take them as follows: upon getting up in the morning he was to take one powder; two hours thereafter another, and eat a very light breakfast, after which he was to take the last powder and report to me. When he arrived at my office I gave him a very small dose of chloretone—say two grains—and proceeded to take my impressions, as I have stated, without the least trouble. The man will sing my praises for doing what so many failed to do, and which they could have done had they only used chloretone. To any one who may ask, I would be glad to recount my experiences in other cases in which I have used this most important compound.—A. E. Franklin, Dental Register.

